# Linear Graphene Nanocomposite Synthesis and an Analytical Application for the Amino Acid Detection of *Camellia nitidissima* Chi Seeds

**DOI:** 10.3390/ma10040443

**Published:** 2017-04-24

**Authors:** Jinsheng Cheng, Ruimin Zhong, Jiajian Lin, Jianhua Zhu, Weihong Wan, Xinyan Chen

**Affiliations:** 1 School of Ying-Dong Food Sciences and Engineering, Shaoguan University, Shaoguan 512005, China; linjiajian@yahoo.com (J.J.L); jhuazh@163.com (J.H.Z); weihonggd@gmail.com (W.H.W); 2 Soochow Tanfeng Graphene Technology Co. Ltd., Suzhou 215100, China; 3 School of Medicine, Jiaying University, Meizhou 513031, China; xiny.chen@hotmail.com (X.Y.C)

**Keywords:** linear graphene nanocomposites, stir bar sorptive extraction, amino acid, *Camellia nitidissima* Chi

## Abstract

Husk derived amino modified linear graphene nanocomposites (aLGN) with a diameter range of 80–300 nm and a length range of 100–300 μm were prepared by a modified Hummers method, ammonia treatment, NaBH_4_ reduction and phenylalanine induced assembly processes, etc. The resulting composites were characterized by transmission electron microscopy (TEM), atomic force microscopy (AFM), scanning electron microscopy (SEM), biological microscope (BM), and X-ray diffraction spectroscopy (XRD), etc. Investigations found that the aLGN can serve as the novel coating of stir bar sorptive extraction (SBSE) technology. By combing this technology with gas chromatography–mass spectrometry (GC-MS), the combined SBSE/GC-MS technology with an aLGN coating can detect seventeen kinds of amino acids of *Camellia nitidissima* Chi seeds, including Ala, Gly, Thr, Ser, Val, Leu, Ile, Cys, Pro, Met, Asp, Phe, Glu, Lys, Tyr, His, and Arg. Compared to a conventional polydimethylsiloxane (PDMS) coating, an aLGN coating for SBSE exhibited a better thermal desorption performance, better analytes fragmentation depressing efficiencies, higher peak intensities, and superior amino acid discrimination, leading to a practicable and highly distinguishable method for the variable amino acid detection of *Camellia nitidissima* Chi seeds.

## 1. Introduction

The novel two-dimensional nanomaterial of graphene held significant features of a large surface area, chemical inertness, a strong physical adsorption of organics, and analyte fragmentation depressing properties, etc. [1,2,3,4,5], which meant that it was a good candidate for the absorption or detection of small moelcules [6,7]. Since 2008, a variety of graphene nanocomposites with different morphologies, for example, nanosheets [8], nanobelts [9], quantum dots [10], nanofibers [11], and three-dimensional topographies [12], etc., have been reported. Among which, linear graphene nanocomposites have attracted much attention due to excellent mechanical, electrochemical, and catalytic characteristics [13,14]. Until now, limited reports have concerned the fabrication of phenylalanine induced amino modified linear graphene nanocomposites and corresponding analytical applications for the detection of amino acids.

*Camellia nitidissima* Chi, a kind of plant with beautiful golden flowers found in southern Asia, has a good reputation as the “Giant Panda of Botany” and “Emperor in Theaceae” [15]. It is also included in the Ι-class national protection of wild plants of China. Most research concerns the analytical or separating study of the plant for its medicinal or nutrient ingredients, including the amino acids, vitamins, etc. Most previous research focus on leaves or flowers of *Camellia nitidissima* Chi [16,17]. Few reports have focused on the detection of amino acids of *Camellia nitidissima* Chi seeds.

**Figure 1:**
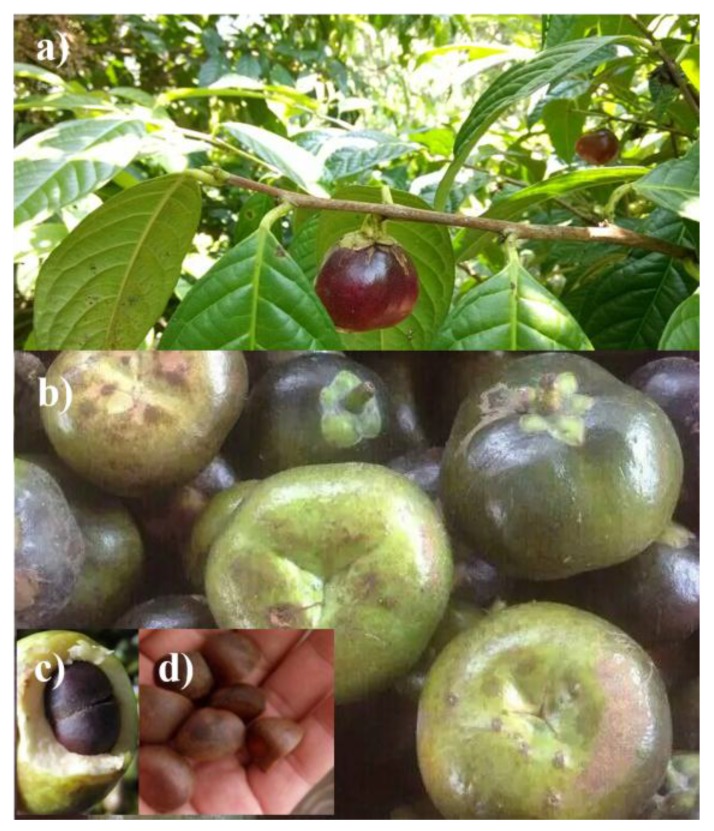
Photos of *Camellia nitidissima* Chi: **a**;**b**) fruit; **c**) fruit flesh; and **d**) seeds.

As illustrated in Figure 1, the *Camellia nitidissima* Chi fruit has a sphere like structure, which has an approximate diameter range of 2–5 cm (Figure 1a,b). An immature fruit has a cyan or green peel, while a mature one displays a brown color. Under the peel is a fruit flesh with a thickness of about 1–1.5 cm (Figure 1c). Inside the fruit flesh are six or seven seeds of the *Camellia nitidissima* Chi plant (Figure 1d).

Normally, automatic amino acid analyzers or chromatogram technologies are widely used analytical methods for the detection of amino acids, although they encounter difficulties when detecting variable kinds of amino acids of *Camellia nitidissima* Chi with a high sensitivity and discrimination [17,18], or even the lack of the detection of proline (Pro), etc. [19]. The development of new suitable and discriminable analytical technologies for amino acids of *Camellia nitidissima* Chi seeds is very important.

The stir bar sorptive extraction (SBSE) technique provides a useful approach for sample enrichment [20], which displayed superior advantages of a high sensitivity, good reproducibility, and outstanding recovery [21]. The coating of conventional stir bars, e.g., polydimethylsiloxane (PDMS) [22] and polyethylene glycol polydimethyl siloxane poly(vinylalcohol) [23], etc., has fragile textures, which would crack or fragment easily in actual analytical experiments [21,23], leading to coating material leftover or matrix interference phenomena in actual detection procedures. Fortunately, due to a high specific surface area, good π–π electrostatic stacking effects, chemical inertness, and a strong Young modulus, etc., a graphene nanocomposite was expected to be a good sorbent material for SBSE technology. Further experiments in our laboratory also confirmed the expectation for the suitability of a SBSE/GC-MS application [24], which were expected to apply in the detection of amino acids of *Camellia nitidissima* Chi seeds.

In this work, husk derived aLGN with a diameter range of 100–300 nm and a length range of 100–300 μm was prepared by a modified Hummers method, ammonia treatment, NaBH_4_ reduction, and phenylalanine induced assembly processes, etc. The resulted composites were characterized in detail, which was used as the coating material of the SBSE for the extraction of amino acids from *Camellia nitidissima* Chi seeds. Under the optimal conditions, an amino acid detection method for* Camellia nitidissima* Chi seeds was proposed based on the combination of SBSE with GC-MS. Compared to the conventional PDMS coating, the aLGN coating showed a better thermal desorption performance, improved analyte fragmentation depressing efficiencies, higher peak intensities, and superior amino acid discrimination, leading to a practicable and highly distinguishable method for variable kinds of amino acid analyses of *Camellia nitidissima* Chi seeds. 

## 2. Results

**Figure 2:**
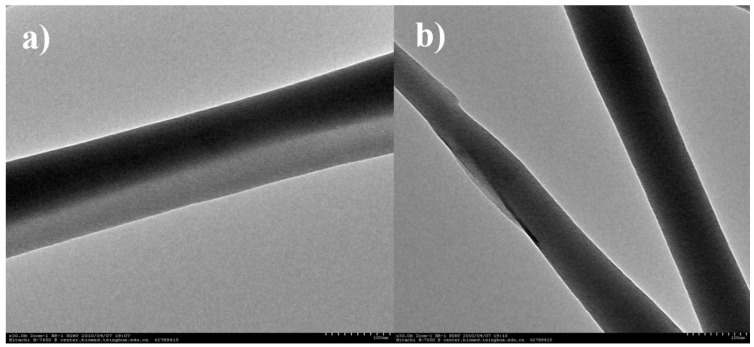
TEM images of (**a**) one piece of aLGN nanocomposites; (**b**) two pieces of aLGN nanocomposites.

**Figure 3:**
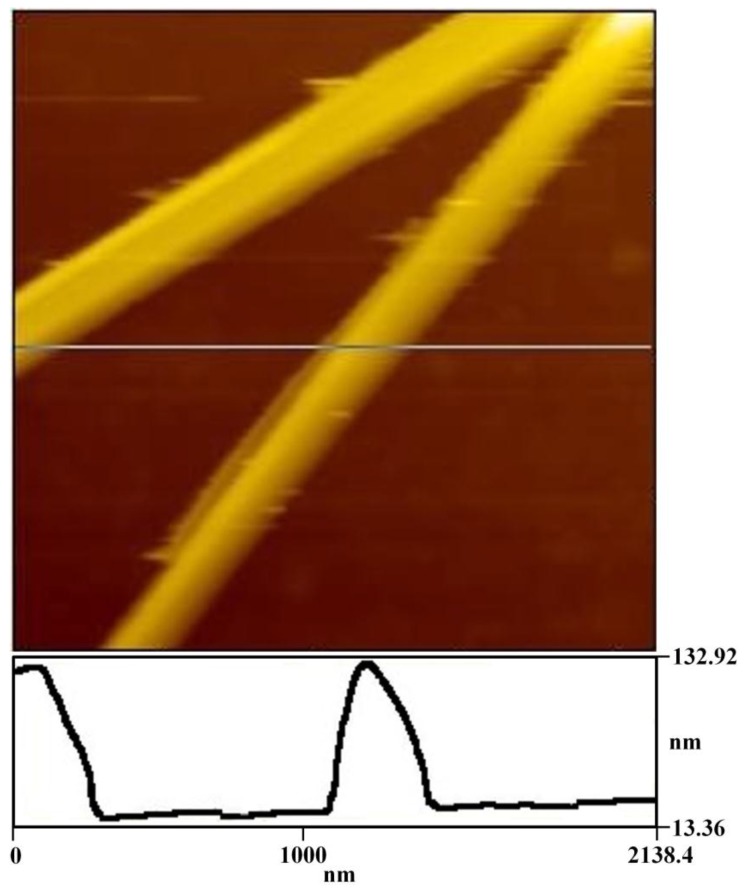
AFM image and depth profile of aLGN on a mica substrate, size 2.138 × 2.138 μm.

The resulting composites were characterized in detail by TEM, AFM, SEM, BM, and XRD, etc. The TEM images illustrated in Figure 2 display a view of the aLGN composites, clearly demonstrating typical linear structured graphene nanocomposites with a diameter of about 100 nm. The AFM results shown in Figure 3 also verified the linear morphologies observed in Figure 2. Furthermore, as illustrated in the depth profile of Figure 3, the diameter of the nanocomposites at the crossed line was about 110 nm, which was in accordance with the TEM results (Figure 2).

**Figure 4:**
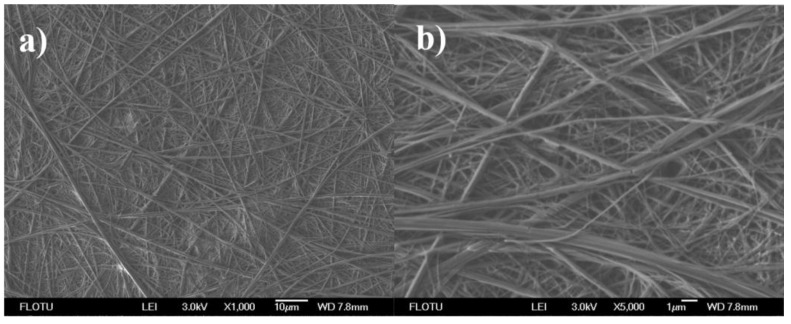
SEM images of aLGN nanocomposites.

The SEM image shown in Figure 4a revealed that the prepared linear graphene nanocomposites had a length of over 100 μm. Moreover, Figure 4b revealed that the aLGN nanocomposites had a diameter range of about 80–300 nm. On the other hand, some linear nanocomposites might combine together, forming nanocomposites with bigger diameters. Further BM observations displayed in Figure S1 found that most aLGN nanocomposites had a length range of about 100–300 μm (Figure S1, see Supplementary Materials).

From the XRD patterns shown in Figure S2 of Supplementary Materials, we could observe that the aLGN nanocomposites gave a characteristic peak at 2θ = 24.7°, which was in conformity with the literature results of the graphene peak (002) [1,25,26,27], confirming that the prepared aLGN nanocomposites still retained the intrinsic attributes of graphene.

**Figure 5:**
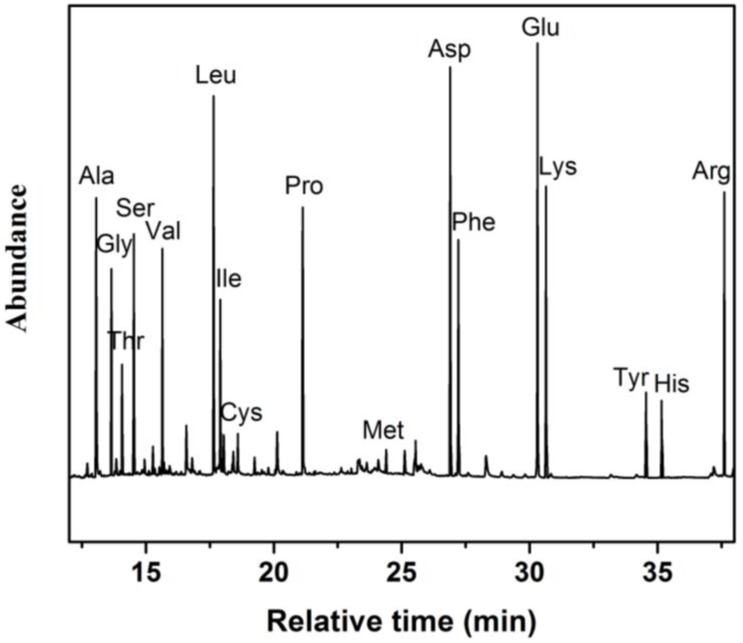
Total ion chromatogram of aLGN coated SBSE/GC-MS analysis for *Camellia Nitidissima* Chi seeds.

According to the thermal desorption and GC-MS procedures illustrated in the “Experimental” section, the amino acid ingredients of the *Camellia Nitidissima C*hi seeds can be readily analyzed. Figure 5 shows a total ion chromatogram of an aLGN coated SBSE/GC-MS analysis of the amino acid components in *Camellia Nitidissima* Chi seeds, in which we can observe that seventeen kinds of amino acids (Ala, Gly, Thr, Ser, Val, Leu, Ile, Cys, Pro, Met, Asp, Phe, Glu, Lys, Tyr, His, and Arg) were detected with high peak intensities and a good discrimination, including seven kinds of essential amino acids (EAAs, Thr, Val, Leu, Ile, Met, Phe, and Lys). 

As illustrated in Table 1, the most abundant amino acid was glutamate (Glu, 7.5), contributing 11.22% of the total amino acid content. Aspartic acid (Asp, 7.0) was the second most abundant amino acid in the *Camellia Nitidissima* Chi seeds, containing 10.48% of the total amino acid content.

## 3. Discussion

### 3.1. Possible Mechanism for the aLGN Formation

**Figure 6:**
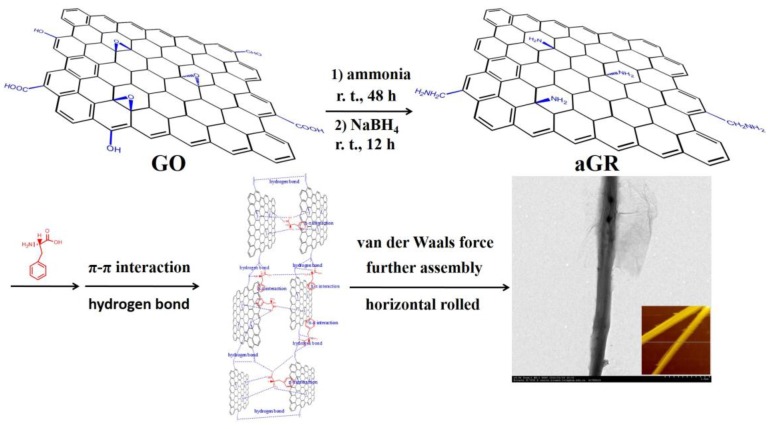
Possible mechanism for the aLGN formation.

In this work, the aLGN nanocomposites were prepared by a modified Hummers method, ammonia treatment, NaBH_4_ reduction, and phenylalanine induced assembly processes, etc. A possible mechanism for the formation of the aLGN nanocomposites can be described by the procedures below: Graphene oxide (GO) was prepared by a modified Hummers method based on the prepared husk derived graphite microcrystalline (see Supplementary Materials). Originally, the husk derived GO showed a 2D nanosheet morphology. Then, the obtained solid was reacted with ammonia for 48 h at room temperature. In this step, ammonia reacted with the epoxy groups and carboxyl groups on the GO nanosheet, producing epoxy-opened structures and amide groups (Figure 6). Through NaBH_4_ reduction in the following step, the reduced amino modified graphene (aGR) was readily obtained.

In the final step, after the addition of phenylalanine, abundant hydrogen bonds between the -NH_2_ groups on the aGR nanosheets and amino groups of phenylalanine were readily formed. Meanwhile, due to the graphene-based special fused aromatic structure, the prepared graphene nanocomposites maintained strong π–π interactions with the benzene rings of the phenylalanine molecules. By synergistic effects with plentiful hydrogen bonds, the synthesized graphene nanosheets were gradually assembled, formed wider and longer graphene nanosheets, and heavily wrinkled or distorted huge graphene nanosheets were also formed. Significantly, due to an increasing number of amino modified graphene nanosheets and phenylalanine molecules involved in the assembly procedures, a strong longitudinal van der Waals force occurred. Induced by the residual phenylalanine (forming more and more hydrogen bonds and π–π interactions, etc., horizontally), further horizontal assembly effects induced the originally formed huge distorted graphene nanosheet to roll gradually (Figure 6), forming the final linear graphene nanocomposites: aLGN. Significantly, from the TEM images of Figure S3a–d in the Supplementary Materials, we could also observe the step-wise, gradual transformation of the two-dimensional graphene nanosheet into the one-dimensional linear graphene nanocomposites.

To further verify the forming mechanism, we carried out a series of experiments to learn about the reactions taking place between aGR and L-phenlyalanine. As shown in Figure S4c (SEM image) and Figure S4f (TEM image) in the Supplementary Materials, with the addition of L-Phe (0.025 mg/mL, 6 mL), linear graphene nanocomposites can be observed. From the TEM image shown in Figure S4f, we can observe a residual two-dimensional graphene nanosheet twining around the linear graphene nanocomposites. On the contrary, in the absence of L-Phe, the aGR nanocomposites maintain a very wrinkled two-dimensional morphology (Figure S4a) instead of a one-dimensional linear pattern, indicating that L-Phe addition is very important for the preparation of linear graphene nanocomposites. It’s interesting that an insufficient addition of L-Phe led to incomplete linear graphene nanocomposites (Figure S4e). Detailed efforts found that the optimal L-Phe addition amount was 6 mL, with a concentration of 0.025 mg/mL. Under such a condition, the prepared graphene nanocomposites with a linear morphology were achieved (Figure S4c,f).

To identify the main driving force forming the prepared one-dimensional linear graphene nanocomposites, we chose a-hexylthiophene (CAS 18794-77-9) instead of L-Phe to induce similar experimental procedures. The structure of the former choice has no aromatic ring or oxygen atoms (or nitrogen, fluorine atoms), therefore, no hydrogen bonds or π–π interactions can form between the a-hexylthiophene molecules and aGR nanosheets. As illustrated in Figure S4b, we could observe that the synthesized nanocomposites showed a wrinkled two-dimensional morphology instead of a one-dimensional linear pattern, indicating that hydrogen bonds or π–π interactions was key element for forming the target one-dimensional linear graphene nanocomposites. Furthermore, 2, 6-diisopropylnaphthalene (CAS 24157-81-1) induced aGR assembly processes were also studied in our laboratory. A 2, 6-diisopropylnaphthalene molecule has an aromatic structure (similar to aGR), which contains no O, N orF atoms. Therefore, few hydrogen bonds can form in such 2, 6-diisopropylnaphthalene induced synthetic processes. The main driving force between the 2, 6-diisopropylnaphthalene molecules and aGR nanosheets was π–π interactions instead of hydrogen bonds. From Figure S4d of the Supplementary Materials, we can observe incomplete and discrete linear graphene nanocomposites, indicating that π–π interactions were one of the main driving forces, which could lead to the formation of the desired linear graphene nanocomposites. However, as illustrated in Figure S4d, only sole kind of π–π interactions as the driving force could not lead to the successful formation of the prepared linear graphene nanocomposites. From the other results shown in Figure S4b,c, etc., we could conclude that hydrogen bonds might be another important driving force forming the target linear graphene nanocomposites.

The proposed hydrogen bond and π–π interaction induced mechanisms or small molecule induced assembly procedures forming a similar nanostructure were also conjectured by the literature. For example, Zhao et al. prepared single crystalline submicrotubes of a small organic functional molecule, 2, 4, 5-triphenylimidazole (TPI, containing N atoms and aromatic rings). The driving forces which form the target nanotube nanocomposites were the H-bonds (horizontal) and interactions (inward), together with the van de Waals force (vertical) [28]. Han et al. demonstrated a straightforward peptide-graphene hybrid assembly into core-hell nanowires. The resultant core/shell nanowires were prepared by peptide (diphenylalanine, containing aromatic rings and N atoms) induced assembly procedures [29].

### 3.2. aLGN Coating Based SBSE/GC-MS Detection for Camellia Nitidissima Chi Seeds

*Camellia nitidissima* Chi fruit was collected carefully, according to Chinese Pharmacopoeia 2015 edition. Seeds were separated, washed and air dried, before being pounded to pieces. The sample was extracted by a Soxhlet extractor with ethanol, producing an extracted sample of *Camellia nitidissima* Chi seeds. A sample SBSE extraction was performed by using an annular tube. Then, a stir bar coated with aLGN was placed on a stainless steel hook, which was immersed in the extracted solution of the *Camellia nitidissima* Chi seeds under stirring. After extraction, the stir bar was introduced into a glass thermal desorption tube (4mm × 187 mm, internal diameter × length). A Frontier PY-2020S pyrolyzer was used in this work. Typically, the desorption temperature was controlled at about 150–300 °C (see Supplementary Materials). 

In this study, by starting from a carefully treated starting material (m_g_), the amino acid complex was extracted according to a modified previous report [30]. By extraction, using precipitation and purification procedures, the extracted amino acid complex (brown solid, m_s_, see Supplementary Materials) was obtained. The extracted m_s_/m_g _ratio and peak abundance for each amino were used for approximating the relative content of each kind of amino acid. Although the extraction or desorption may have been non-uniform for different kinds of amino acids extracted by the aLGN coated stir rod, the final relative ratio for each amino acid had no close connection with the extraction and desorption rates. Further research on the extraction and desorption efficiency, together with the extraction selectivity for each amino acid of the *Camellia nitidissima* Chi seeds, are in progresses in our laboratory. 

The contents of Histidine (His), Methionine (Met), and Cysteine (Cys) held the three lowest quantities among all the amino acids. Meanwhile, seven kinds of EAAs (Thr, Val, Leu, Ile, Met, Phe, and Lys) comprised 38.62% of all the amino acid contents. Significantly, as we can see from Figure 5, coating material interference was completely eliminated. Moreover, few fragment ion peaks of the analytes and coating material were observed, indicating that aLGN was a stable coating material for the SBSE extraction of amino acids from *Camellia nitidissima* Chi seeds.

For comparison, we also used conventional PDMS as the novel coating material of SBSE/GC-MS. Only fourteen kinds of amino acids (instead of the seventeen shown in Figure 5) from the *Camellia nitidissima* Chi seeds were detected, including Ala, Gly, Thr, Ser, Val, Leu, Ile, Pro, Asp, Phe, Glu, Lys, His, and Arg. The peaks for Cys, Met, and Tyr shown in Figure 5 were missing (Figure S5, see Supplementary Materials). 

What’s more, when PDMS was serving as the coating material of SBSE instead of aLGN, the most abundant amino acids were Leucine (Leu, 6.5) and aspartic acid (Asp, 6.5) instead of Glu, as illustrated in Table S1. Both Leu and Asp contributed 11.17% of the total amino acid content (see Supplementary Materials). The contents of histidine (His), threonine (Thr), and isoleucine (Ile) held the lowest three quantities among all the amino acids. Meanwhile, only six kinds of EAAs (Thr, Val, Leu, Ile, Phe, and Lys) were found (Table S1) instead of the seven ones shown in Table 1, comprising 40.03% of all the amino acid contents. More significantly, as illustrated in Figure S5, strong background interferences were present when PDMS was used as the SBSE coating material, which greatly obscured the detection performance of the amino acids of *Camellia nitidissima* Chi seeds.

Meanwhile, as shown in Table 1, when aLGN served as the SBSE coating material, the total relative content of all the detected amino acids was 66.8 (including 17 kinds of amino acids). While when the SBSE coating material was replaced by PDMS, the total relative content of all the detected amino acids changed to 58.2 (including 14 kinds of amino acids) with serious background interferences. The experimental results indicates that aLGN held better thermal desorption efficiencies and coating stability for the amino acids of *Camellia nitidissima* Chi seeds than PDMS when serving as the SBSE coating material. Therefore, aLGN nanocomposite coated SBSE/GC-MS can display high thermal desorption efficiencies and coating stability for the amino acids of *Camellia nitidissima* Chi seeds without coating material or analyte interference, providing a novel and highly distinguishable method to determine the different amino acid ingredients of *Camellia nitidissima* Chi seeds.

Some recent literature also revealed that conventional commercial PDMS had deficiencies or a poor performance when serving as solid phase microextraction (SBSE etc.) coating material in analytical procedures. For example, in recent years, Ochiai et al. developed a novel solvent-assisted stir bar sorptive extraction (SA-SBSE) technique. In combination with different GC-MS technologies, aroma compounds in beer and pesticides in wine were readily detected. This work used solvent swollen PDMS to replace commercial PDMS for the SBSE coating. A better analytical performance for aroma compounds or pesticide detection was obtained when compared with an untreated commercial PDMS coating [31]. Wei *et al*. extracted the volatile components in ripening fruit peel and pulp of mango (*Mangifera indica* L. var Zihua) with 100 μm PDMS or 75 μm Carboxen/ PDMS fibres, prior to GC-MS analysis. By using 75 μm Carboxen/PDMS fibre as the coating material, 26 and 21 kinds of volatile components were identified in mango peel and pulp, respectively. However, when commercial 100 μm PDMS fibre was used, only 22 volatile components were identified in mango peel, together with 15 kinds of volatile components in the mango pulp. Therefore, 75 μm Carboxen/PDMS fibre provided a much more suitable tool for the extraction of volatile components in mango fruits and the peel when compared with a conventional PDMS coating [32]. Cai et al. developed a novel dibenzo-18-crown-6 as a coating for solid phase microextraction, and by combining it with GC-FID, the extraction properties of the new coating for derivatized aliphatic amines such as methylamine and dimethylamine, etc., were investigated. A further investigation found that the novel dibenzo-18-crown-6 coating had a better extraction performance in the headspace extraction of a group of tetrafluorobenzoic acid N-hydroxysuccinimide ester amine derivatives than the commercial PDMS coating. The authors believed that the dibenzo-18-crown-6 coating provided much better results than the commercial PDMS coating because the benzyl rings favour π–π interactions, and the greater number of oxygen atoms in the crown-ether ring favour polar interactions [33]. Such effects were similar to the aLGN nanocomposites prepared in this work, as the prepared amino modified aLGN nanocomposites possess strong π–π interactions and polar interactions for further SBSE extraction of amino acids.

## 4. Materials and Methods 

### 4.1. Chemicals

Husk was collected in Shaoguan city, Guangdong, China. *Camellia nitidissima* Chi seeds were collected from Fangcheng port city, Guangxi Zhuang Autonomous Region, China. L-phenylalanine, dicyclohexylcarbodiimide (DCC), N-hydroxysulfosuccinimide sodium salt (Sulfo-NHS), and N, N-dimethylformamide (DMF) were purchased from Alfa Aesar China (Tianjin) Co. Ltd. (Tianjin, China). The water used in this work was triple distilled water or deionized water. All solvents and other reagents were purchased from Beijing Chemicals Co. Ltd. (Beijing, China).as analytical-grade products. 

### 4.2. Apparatus and Methods

The polydimethylsiloxane (PDMS) coated stir rod (10 mm × 3.2 mm, length × outside diameter) and thermal desorption tube (4mm × 187 mm, internal diameter × length) were provided by Gerstel Co. Ltd., Germany. The carboxyl functionalized stir rod without coating (10 mm × 2.4 mm, length × outside diameter, iron core inside), glass vial (sample annular tube, 12 mL), and stainless steel hook (15 cm) were provided by Soochow Tanfeng company, China. JEOL JEM 1200EX and JEOL JEM 2010 transition electronic microscopy (Jeol Datum Ltd., Tokyo, Japan) were used for transmission electron microscopy (TEM) analysis at an accelerating voltage of 100 kV. Samples were prepared by placing one drop of an ethanol suspension of the aLGN nanomaterial onto a copper grid (3 mm, 200 mesh) coated with a carbon film. JSM-7401 scanning electron microscopy (SEM) (Joel Datum, Tokyo, Japan) operated at 20 kV was used to analyze the sample. Atomic force microscopic (AFM) (Digital Instruments Inc., Austyn, USA) images were taken using a Nanoscope III Multi Mode SPM with an AS-12 (“E”) scanner operated in tapping mode in conjunction with a V shaped tapping tip (Applied Nano-structures SPM model: ACTA). The images were taken at a scan rate of 2 Hz. Biological microscope (BM) ( Olympus (China) Co., Ltd., Beijing, China) observations were conducted on an OLYMPUS CX23 biological microscope. The powder X-ray diffraction (XRD) (Bruker Corporation, Billerica, USA) measurements of the samples were recorded on a Bruker D8- Advance X-ray powder diffractometer using Cu Kα radiation (λ = 1.5406 Å) with scattering angles (2θ) of 8°–60°.

The aLGN coated SBSE/GC-MS spectrometer in EI mode was performed using a Shimadzu GCMS-QP2010 Plus system (Shimadzu, Kyoto, Japan). Data acquisition and analysis were performed using software from GC-MS Solution (Shimadzu, Kyoto, Japan). The separation procedure was conducted on a PE-5 capillary column (30 m × 0.25 mm × 0.25 μm). For thermal desorption, a Frontier PY-2020S pyrolyzer (Frontier Co. Ltd., Okayama, Japan) was used in this work. The thermal desorption unit was mounted on Shimadzu GCMS-QP2010 Plus equipped with a CIS 4 PTV inlet. The desorbed solutes were cryofocussed in the PTV inlet. After desorption, the PTV was programmed to inject the solutes and the compounds were analyzed on a capillary column. The oven temperature was programmed at 70 °C for 2.0 min, increased to 190 °C at a rate of 15 °C/min and insulated for another 1.0 min, before being increased to 260 °C at a rate of 10 °C min^−1^. Finally, the temperature was increased to 285 °C at a rate of 5 °C/ min, and then insulated for 10.0 min. The cut time of the solvent was 4.5 min. A splitless injection mode was used (splitless time: 1.0 min) and the injection volume was 1.0 mL. High purity Helium (99.999%) was used as the carrier gas, with a flow rate of 1.20 mL/min. The injection port, interface temperatures, and ion source and were 290, 280, and 220 °C, respectively. The National Institute of Standards and Technology’s (NIST) mass spectral database was used in this work. All experiments were performed in triplicate.

### 4.3. aLGN Nanocomposites Synthesis

Husk derived graphite microcrystalline was prepared according to our previous work [34] (see Supplementary Materials). By starting from the prepared husk derived graphite microcrystalline, GO was prepared by a modified Hummers method based on previous work [4,5,35] (see Supplementary Materials). The obtained GO (100 mg) was suspended in water (100 mL), and the mixture was then sonicated for 1 h. Successively, ammonia (9.65 mL) was added slowly to the mixture under stirring, the mixture was then sonicated for another 2 min, and was then stirred for 48 h under room temperature. After being filtrated, washed, and vacuum-dried at 40 °C for 24 h, a black solid was readily produced. The obtained solid was re-suspended in water forming a dispersion of 1 mg/mL, and NaBH_4_ (750 mg) was then gradually added to the mixture. The suspension was stirred at 80 °C for 4 h. Finally, the dispersion was cooled down and filtered, and the solid sample was collected after thorough washing with ethanol and deionized water until the impurities were completely removed. The sample was vacuum-dried at 35 °C for 24 h, affording the reduced amino modified composite: aGR. Finally, a dispersion of aGR (0.1 mg/mL, 100 mL) was prepared, and the L-phenylalanine solution (0.025 mg/mL, 6 mL) was slowly added under stirring at room temperature. After the addition, the mixture was stirred for another 4h at room temperature. The mixture was filtered, washed, and vacuum-dried at 35 °C for 24 h, affording the final nanocomposites: aLGN. 

## 5. Conclusions

In summary, husk derived aLGN with a diameter range of 100–300 nm and a length range of 100–300 μm was prepared by a modified Hummers method, ammonia treatment, NaBH_4_ reduction, and phenylalanine induced assembly processes, etc. The resulting composites were characterized in detail. The prepared aLGN composite was used as an extraction coating material of SBSE for amino acids of *Camellia nitidissima* Chi seeds. Under the optimal conditions, an amino acid detection method for *Camellia nitidissima* Chi seeds was proposed, based on aLGN coated SBSE/GC-MS technology, which can readily detect seventeen variable kinds of amino acids of *Camellia nitidissima* Chi seeds, including Ala, Gly, Thr, Ser, Val, Leu, Ile, Cys, Pro, Met, Asp, Phe, Glu, Lys, Tyr, His, and Arg. Compared to the conventional PDMS coating material, the aLGN coating exhibited a better thermal desorption performance, greater analyte fragmentation depressing efficiencies, higher peak intensities, and superior amino acid discrimination, providing a practicable and highly distinguishable method for variable kinds of amino acid analyses of *Camellia nitidissima* Chi seeds. The use of renewable husk as the new carbon source of the aLGN led to a comprehensive utilization of renewable resources andlow cost graphene nanocomposite synthesis.

## Figures and Tables

**Table 1:** aLGN coated SBSE/GC-MS analysis of *Camellia Nitidissima* Chi seeds (n = 3 ^1^).

Entry	Amino Acids (Retention Time/min)	Relative Content ^2^ (mg/g Dry Wet)
1 2 3 4 5 6 7 8 9 10 11 12 13 14 15 16 17 18 19	Ala (13.06) Gly (13.65) Thr ^3^ (14.07) Ser (14.52) Val ^3^ (15.64) Leu ^3^ (17.65) Ile ^3^ (17.91) Cys (18.59) Pro (21.14) Met ^3^ (24.39) Asp (26.90) Phe ^3^ (27.21) Glu (30.31) Lys ^3^ (30.64) Tyr (34.55) His (35.16) Arg (37.61) EAAs Total	4.9 3.7 2.1 4.3 4.1 6.6 3.2 0.6 4.8 0.5 7.0 4.2 7.5 5.1 1.7 1.5 5.0 25.8 66.8

^1^ All experiments were performed in triplicate; ^2^ Relative content (%) = relative abundance (%, in Figure 5) × m_s_/m_g_; ^3^ EAAs.

## Supplementary Materials

The following are available online at www.mdpi.com/1996-1944/10/4/443/s1: Figure S1: Biological microscope of aLGN nanocomposites; Figure S2: XRD patterns of aLGN nanocomposites; Figure S3: Verification experiments for aLGN; Figure S4: TEM images for aLGN forming evidences; Figure S5: Total ion chromatogram of PDMS coated SBSE/GC-MS analysis for Camellia nitidissima Chi seeds; Table S1: PDMS coated SBSE/GC-MS analysis of Camellia nitidissima Chi seeds; Materials and methods: husk derived graphite microcrystalline preparation; GO synthesis based on husk derived graphite microcrystalline; preparation of aLGN coated stir rod, free amino acids extraction of Camellia nitidissima Chi seeds, sample extraction, and desorption procedures, etc.
