# Supplementary material for: Linear Graphene Nanocomposite Synthesis and an Analytical Application for the Amino Acid Detection of Camellia nitidissima Chi Seeds
Source: Materials (Basel). 2017 Apr 24;10(4):443. doi: 10.3390/ma10040443 (PMC5506894; doi:10.3390/ma10040443)
Supplement: Supplementary file 1 [file materials-10-00443-s001.pdf]

Supplementary Materials: Linear Graphene Nanocomposites Synthesis and Analytical Application for Amino Acids Detection of *Camellia Nitidissima* Chi Seeds

Jinsheng Cheng [1,2,*], Ruimin Zhong [1,*], Jiajian Lin [1], Jianhua Zhu [1], Weihong Wan [1] and Xinyan Chen [3]

1. Materials and Method

1.1. Husk Derived Graphite Microcrystalline Preparation

As reported in our previous work [1]: Careful collected husks were immersed in water, the floating husks on the surface of the water were removed. After filtration, the preferable selected husks were washed with water for 3 times and vacuum-dried. Then the treated husks (10 g) were smashed and sieved with 30 mesh sieve. The obtained husk powder was placed in a clean porcelain crucible. Subsequently, zinc chloride solution (0.05 mol/L, 10 mL) was added as the activator. The mixture was stirred for several mins, which was kept wet impregnation at 25 °C for 12 h. The treated sample was then placed into a muffle furnace, the temperature was raised from room temperature to 900 °C (heating rate: 10 °C/min). After thermal insulation for 1 h, the activated sample were taken out from the muffle furnace, which was then added to muriatic acid (0.1 mol/L) immediately. The sample was then filtered and washed with deionized water for several times to pH neutral. The sample powder was dried at 110 °C for 4 h, then naturally cooled in a desiccator. Finally, the sample was smashed again together with sieving treatment with 300 mesh screen, affording the husk derived crude activated carbon. The crude solid was dialyzed (Cellu. sep., 6000–8000) in deionized water (pH 7.38) for 1 week, then the sample was vacuum-dried (0.09 MPa) at 50 °C, giving the refined activated carbon.

The prepared activated carbon contained graphitized graphite microcrystalline and non-graphitized amorphous activated carbon. Based on our previous work [1], we tried to used anaerobic high temperature heat treatment technologies to make the amorphous carbonaceous activated carbon graphitized completely, forming graphite microcrystalline. The detailed procedures showed below: The prepared activated carbon (10 g) and Fe_2O_3 catalyst (0.4 g) was placed in a quartz boat. The thermal treatment was carried out under argon atmosphere (99.99%). The temperature of the system was raised to 1800 °C with a heating rate of 45 °C /min. After thermal insulation for 2 h, the treated sample was washed with deionized water and dried at 110 °C until the water content decline to 9.5%. The dried sample was placed into a pipe furnace under oxygen and nitrogen mixture (the ratio of oxygen to nitrogen was 1:9). Program warming processes were carried out. The system was kept at 450 °C for 10 min, then the temperature was cooled down gradually to 25 °C. The obtained sample was washed with 10% HCl for 3 times to remove the catalyst ions. Then the sample powder was washed with deionized water entirely to remove the acidic impurities. The solid was sieved with 100 mesh screen and vacuum-dried at 60 °C for 24 h, giving the husk derived graphite microcrystalline with a size range of 4~25 μm.

1.2. Graphene Oxide (GO) Synthesis Based on Husk Derived Graphite Microcrystalline

By starting from the husk derived graphite microcrystalline described in 2.1, GO was prepared by modified Hummers method based on previous work [2–6]: husk derived graphite microcrystalline (1.5 g) was put into a mixture of 12 mL concentrated H_2SO_4, 2.5 g $K_2S_2O_8$ and 2.5 g P_2O_5. The solution was heated to 80 °C and kept stirring for 5 h by using oil-bath. In a next step, the mixture was cooled to room temperature and diluted with deionized water (500 mL) overnight. By filtration with 0.2 micron nylon film and drying naturally, a black solid was obtained. The pre-oxidized graphite was then re-oxidized by Hummers method. Pretreated graphite powder was put into 0 °C concentrated

H_2SO_4 (120 mL). Then, 15 g $KMnO_4$ was added gradually under stirring and the temperature of the mixture was kept to be below 20 °C by ice-bath. Successively, the mixture was stirred at 35 °C for 4 h, and then diluted with 250 mL deionized water by keeping the temperature under 50 °C. 700 mL deionized water was then injected into the mixture followed by adding 20 mL 30% H_2O_2 drop by drop. The mixture was filtered and washed with 1:10 HCl aqueous solution (1 L) to remove metal ions followed by 1 L of deionized water to remove the acid. The resulting solid was dried in air and diluted to make graphene oxide dispersion (0.5% w/w). Finally, it was purified by dialysis for one week to remove the remaining metal species, finally, the product was obtained by filtration and dried in vacuum for 24 h at 60 °C.

1.3. Preparation of aLGN Coated Stir Rod

In a reaction flask, the obtained aLGN (100 mg) was suspended in DMF (100 mL), which was ultrasonicated for 30 mins, forming a brown dispersion. Commercially purchased carboxyl functionalized stir rod without coating (10 mm × 2.4 mm, length × outside diameter, iron core inside) was immersed in the aLGN dispersion. In a successive step, 100 mg DCC and 65 mg Sulfo-NHS were added into the flask, followed by stirring treatment for 24 h [6]. Then the stir rod was picked out and washed with ethanol for three times to remove un-coated aLGN. After air-drying under a dust cover, an aLGN coated stir rod was obtained. The thickness of the coating was examined by SEM technology.

1.4. Free Amino Acids Extraction of Camellia Nitidissima Chi Seeds

Camellia nitidissima Chi seeds was collected carefully according to Chinese Pharmacopoeia 2015 edition, then the seeds were separated, washed and vacuum-dried. In typical procedures,[7] vacuum-dried *Camellia nitidissima* Chi seeds (about 6.0 g) were smashed and sieved with 50 mesh screen, then precision weighed sieved powder (5.0 g) was collected as the starting sample (marked m_g), which was transferred into a 250 mL round flask. In a successively step, 70% ethanol (100 ml) was also added to the round flask, the system was refluxed for 2 h. The mixture was cooled down and filtered, then the residual solid was re-refluxed for another 2 runs. All the filtrates were combined, after bleaching, the mixture was vacuum concentrated to 80 ml. The sediment was filtered and 95% ethanol (250 ml) was added to the filtrate. The precipitation (proteins) was filtered and discarded, the ethanol was removed by rotary evaporator. Then the system was treated by 732 cation exchange resin. The obtained compounds were washed with deionized water until the solid became colorless. After this step, the solid was washed with 5% ammonia, the filtrate was collected carefully, which show coloration effect to ninhydrin. The filtrate was vacuum concentrated and vacuum-dried at 30 °C for 8 h, affording light-brown solid (337 mg, marked m_s), which would be used in further experiments. All experiments were performed in triplicate.

1.5. Sample Extraction and Desorption Procedures

The light-brown solid (337 mg) prepared above was dissolved in DMF (8 ml), which was ultrasonicated for 45 mins, giving amino acids extracting solution of *Camellia nitidissima* Chi seeds. The solution was transferred into a glass vial (an annular tube, 12 mL). Then a stir bar coated with aLGN or PDMS was placed on a stainless steel hook (15 cm), which were immersed in the solution under stirring. The system was stirred slowly for another 120 mins. After extraction, the stir bar is introduced in a glass thermal desorption tube (4mm × 187mm, internal diameter × length). Then desorption temperature was controlled at about 150–300 °C during 10 mins.

2. Supporting experimental results

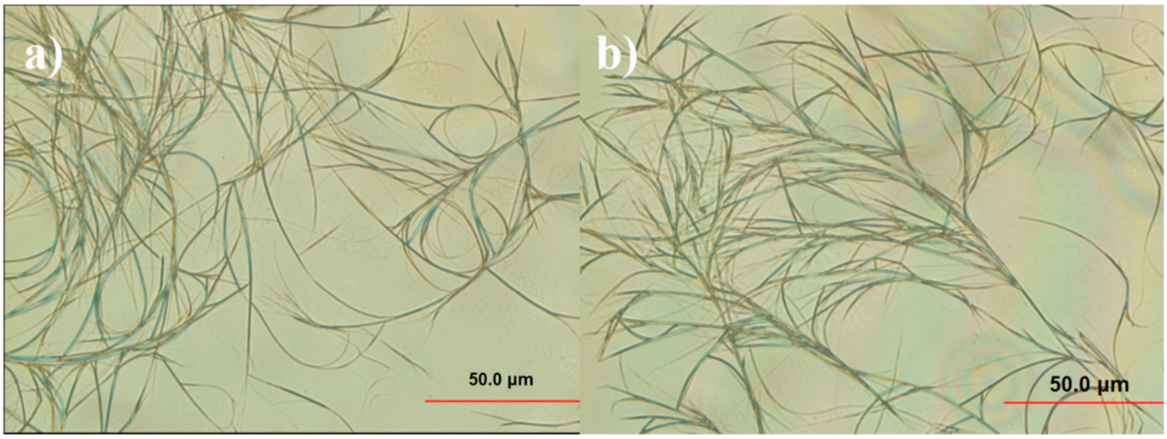

Figure S1. Biological microscope of aLGN nanocomposites.

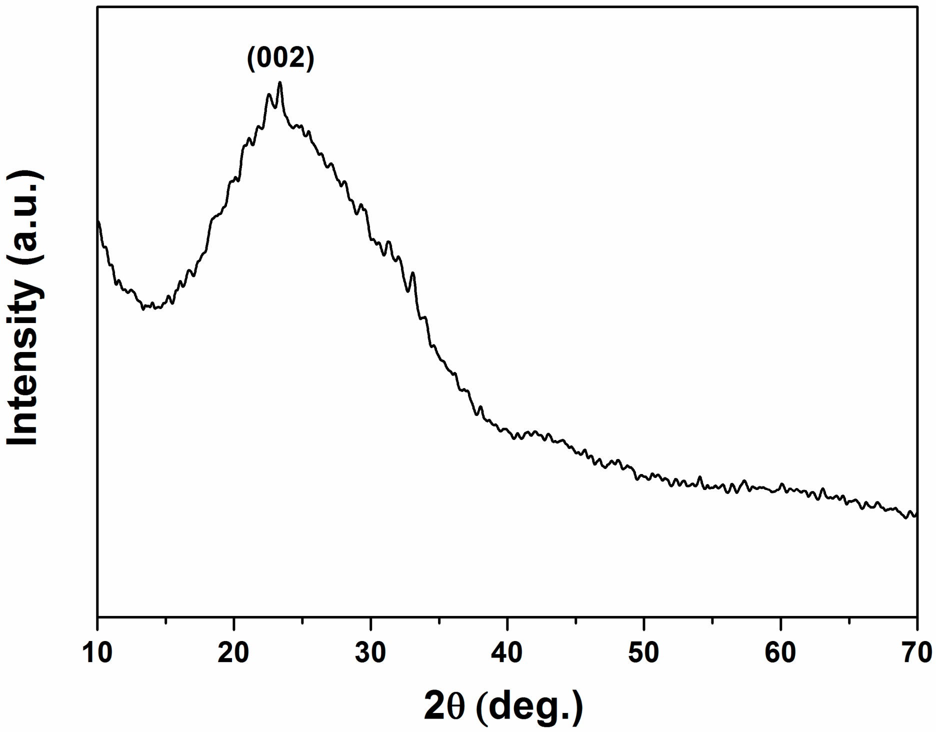

Figure S2. XRD patterns of aLGN nanocomposites.

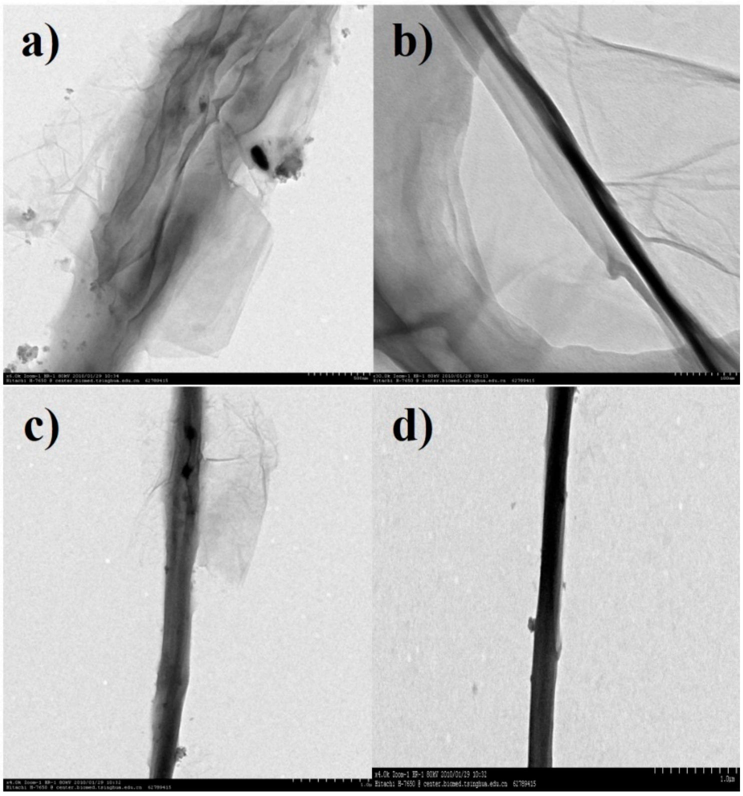

Figure S3. TEM images for aLGN forming evidences (L-Phe, 0.025 mg/mL, 6 mL).

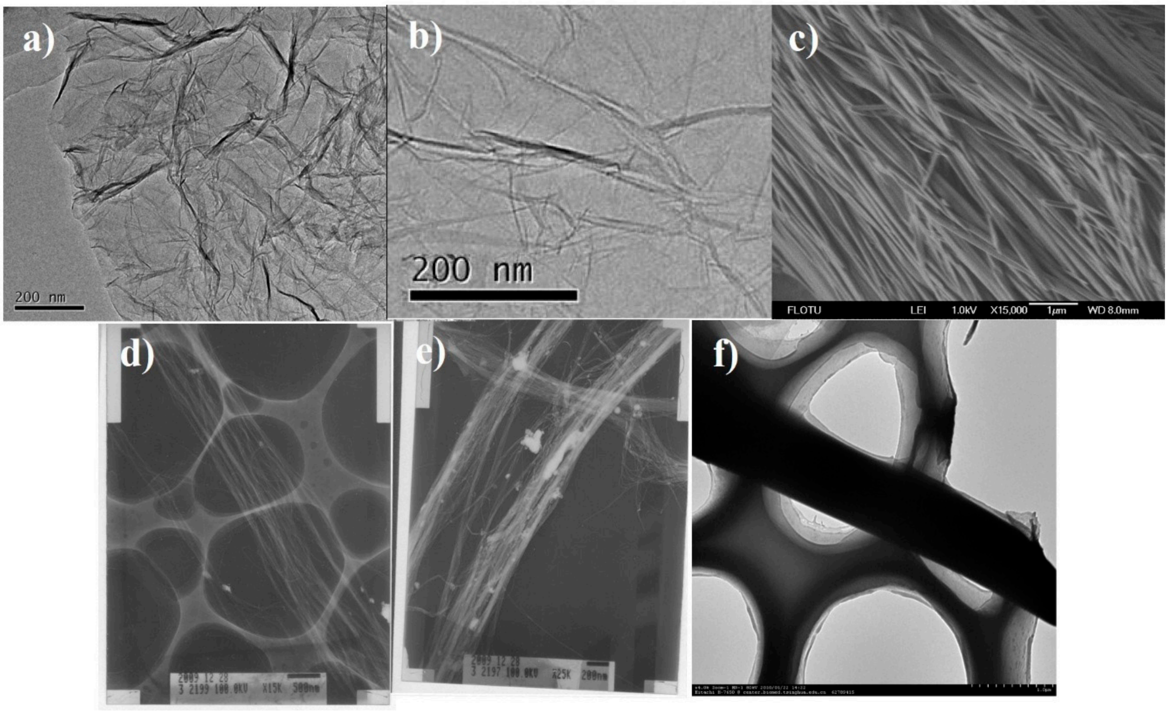

Figure S4. Verification experiments for aLGN formation. **a**) TEM image of the graphene nanocomposites in the absence of L-Phe; **b**) TEM image of the graphene nanocomposites in the presence of a-hexylthiophene (CAS18794-77-9, 0.025 mg/mL, 6 mL); **c**) SEM image of the graphene nanocomposites in the presence of L-Phe (0.025 mg/mL, 2 mL); d) TEM image of the graphene nanocomposites in the presence of 2,6-diisopropylnaphthalene (CAS 24157-81-1, 0.025 mg/mL, 6 mL); e) TEM image of the graphene nanocomposites in the presence of L-Phe (0.025 mg/mL, 2 mL); f) TEM image of the graphene nanocomposites in the presence of L-Phe (0.025 mg/mL, 6 mL).

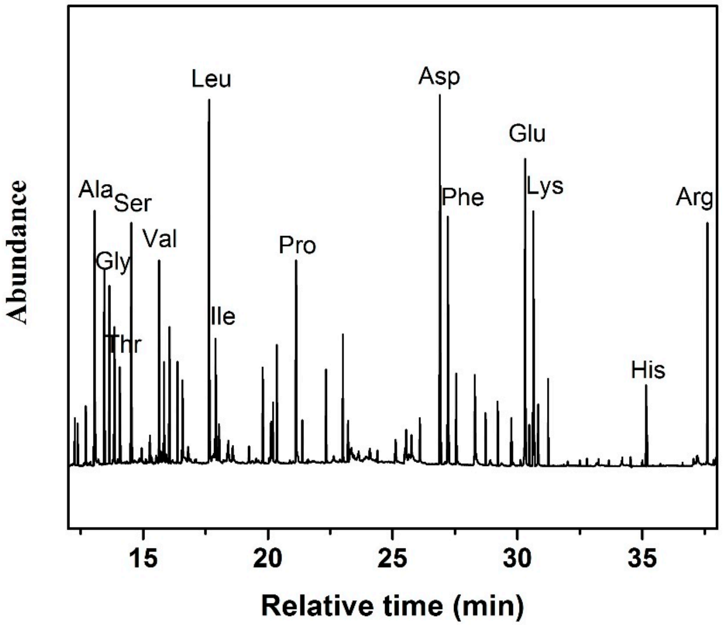

Figure S5. Total ion chromatogram of PDMS coated SBSE/GC-MS analysis for *Camellia nitidissima* Chi seeds.

Table S1. PDMS coated SBSE/GC-MS analysis of *Camellia Nitidissima* Chi seeds (n=3 [1])

Entry	Amino acids (Relative time/min)	Relative content [2] （mg/kg dry wet）
1	Ala (13.06)	4.6
2	Gly (13.65)	3.3
3	Thr[3] (14.07)	1.9
4	Ser (14.52)	4.4
5	Val[3] (15.64)	3.8
6	Leu[3] (17.65)	6.5
7	Ile[3] (17.91)	2.4
8	Pro (21.14)	3.8
9	Asp (26.90)	6.5
10	Phe[3] (27.21)	4.5
11	Glu (30.31)	5.5
12	Lys[3] (30.64)	4.6
13	His (35.16)	1.6
14	Arg (37.61)	4.5
15	EAAs	23.3
16	Total	58.2

[1] All experiments were performed in triplicate; [2]Relative content (%) =relative abundance (%, in Fig. S5) × m_s/m_g; [3] EAAs

References

1. Cheng, J.S.; Wan, W.H.; Chen, X.Y.; Zheng, Q.X. Preparation and structural characterization of graphene by rice husk. *Transact. CSAE* **2015**, *31*, 88–294.
2. Hummers, W.; Offeman, R. Preparation of graphitic oxide. *J. Am. Chem. Soc.* **1958**, *80*, 339–1339.
3. Cheng, J.; Wan, W.; Zhu, W. One-pot solvothermal synthesis of TiO_2 nanobelt/graphene composites for selective renal cancer cells destruction. *Chin. J. Chem.* **2016**, *34*, 53–58.

4. Cheng, J.S.; Tang, L.H.; Li, J.H. Palladium nanoparticles-decorated graphene nanosheets as highly regioselective catalyst for cyclotrimerization reaction. *J. Nanosci. Nanotech.* **2011**, *11*, 5159–5168.
5. Cheng, J.S.; Zhang, G.C.; Du, J.; Tang, L.H.; Xu, J.Y.; Li, J.H. New role of graphene oxide as active hydrogen donor in the recyclable palladium nanoparticles catalyzed ullmann reaction in environmental friendly ionic liquid/supercritical carbon dioxide system. *J. Mater. Chem.* **2011**, *21*, 3485–3494.
6. Yang, X.; Wang, Y.; Huang, X.; Ma, Y.F.; Huang, Y.; Yang, R.C.; Duan, H.Q.; Chen, Y.S. Multi-functionalized graphene oxide based anticancer drug-carrier with dual-targeting function and pH-sensitivity. *J. Mater. Chem.* **2011**, *21*, 3448–3454.
7. Chen, Z.; Liu, Q.; Geng, Z.; Tang, G. Extraction-separation and determination of amino acid in seeds of *Rubia Cordifolia* L. *J. Shanxi Norm. Univ. (Nat. Sci. Ed.)* **1996**, *12*, 118–119.
